# The impact of migration on tuberculosis epidemiology and control in high-income countries: a review

**DOI:** 10.1186/s12916-016-0595-5

**Published:** 2016-03-23

**Authors:** Manish Pareek, Christina Greenaway, Teymur Noori, Jose Munoz, Dominik Zenner

**Affiliations:** Department of Infection, Immunity and Inflammation, University of Leicester, Leicester, UK; Department of Infection and HIV Medicine, University Hospitals of Leicester NHS Trust, Leicester, UK; Division of Infectious Diseases and Department of Clinical Epidemiology, Jewish General Hospital, McGill University, Montreal, Canada; European Centre for Disease Prevention and Control, Solna, Sweden; Barcelona Institute for Global Health, Barcelona, Spain; Centre for Infectious Disease Surveillance and Control, Public Health England, London, UK; Centre for Infectious Disease Epidemiology, University College London, London, UK

**Keywords:** Migration, Tuberculosis, Screening, Review

## Abstract

Tuberculosis (TB) causes significant morbidity and mortality in high-income countries with foreign-born individuals bearing a disproportionate burden of the overall TB case burden in these countries. In this review of tuberculosis and migration we discuss the impact of migration on the epidemiology of TB in low burden countries, describe the various screening strategies to address this issue, review the yield and cost-effectiveness of these programs and describe the gaps in knowledge as well as possible future solutions.

The reasons for the TB burden in the migrant population are likely to be the reactivation of remotely-acquired latent tuberculosis infection (LTBI) following migration from low/intermediate-income high TB burden settings to high-income, low TB burden countries.

TB control in high-income countries has historically focused on the early identification and treatment of active TB with accompanying contact-tracing. In the face of the TB case-load in migrant populations, however, there is ongoing discussion about how best to identify TB in migrant populations. In general, countries have generally focused on two methods: identification of active TB (either at/post-arrival or increasingly pre-arrival in countries of origin) and secondly, conditionally supported by WHO guidance, through identifying LTBI in migrants from high TB burden countries. Although health-economic analyses have shown that TB control in high income settings would benefit from providing targeted LTBI screening and treatment to certain migrants from high TB burden countries, implementation issues and barriers such as sub-optimal treatment completion will need to be addressed to ensure program efficacy.

## Background

In this review (see Table 1) we first analyse the burden of tuberculosis (TB) in foreign-born, migrant populations before going on to discuss the drivers of the current TB epidemiology in these populations focusing on migration patterns, the importance of reactivation of latent tuberculosis infection as compared to the burden of imported active TB and molecular genotyping data underpinning these studies. We then go on discuss, in detail, the methods, outcomes, and cost-effectiveness of the different TB control strategies in place for migrant populations.

**Table 1:** Key messages about tuberculosis and migration in high-income countries

• Tuberculosis continues to be a public health concern in high-income countries
• Tuberculosis burden in high-income countries is primarily amongst the foreign-born, migrant population
• The reasons underlying this burden are the interaction of migration from high TB burden countries and the reactivation of remotely acquire latent tuberculosis infection in the first five years after arrival
• Genotyping data suggests that there is relatively little transmission in migrant communities in the receiving country
• Methods of TB control in migrant population have historically focused on identifying active tuberculosis but the yields for this remain relatively low
• Screening migrants for latent tuberculosis infection may have a higher yield although implementation may be difficult
• The health economics of screening migrants for active and/or latent tuberculosis is a topic of much debate
• Targeted pre-arrival screening for active TB and post arrival screening for latent tuberculosis infection in migrants from intermediate/high TB burden settings may provide the most cost-effective solution
• Implementation of programmatic screening is limited by uptake, acceptance and completion of therapy

## Tuberculosis epidemiology in high-income countries

Tuberculosis (TB) remains a ‘global health emergency’ [1]. Although much of the burden is concentrated in high-burden settings in Asia and Africa (which make up 58 % and 28 % of all cases respectively) [2], TB continues to be of concern in high-income nations. In the 34 high-income Organisation for Economic Cooperation and Development (OECD) countries, TB incidence fell by a median of 4.7 % per year (between 1995 and 2004) decelerating to 3.0 % per year between 2005 and 2014 [3] – making TB elimination more difficult to attain [4].

**Fig. 1:**
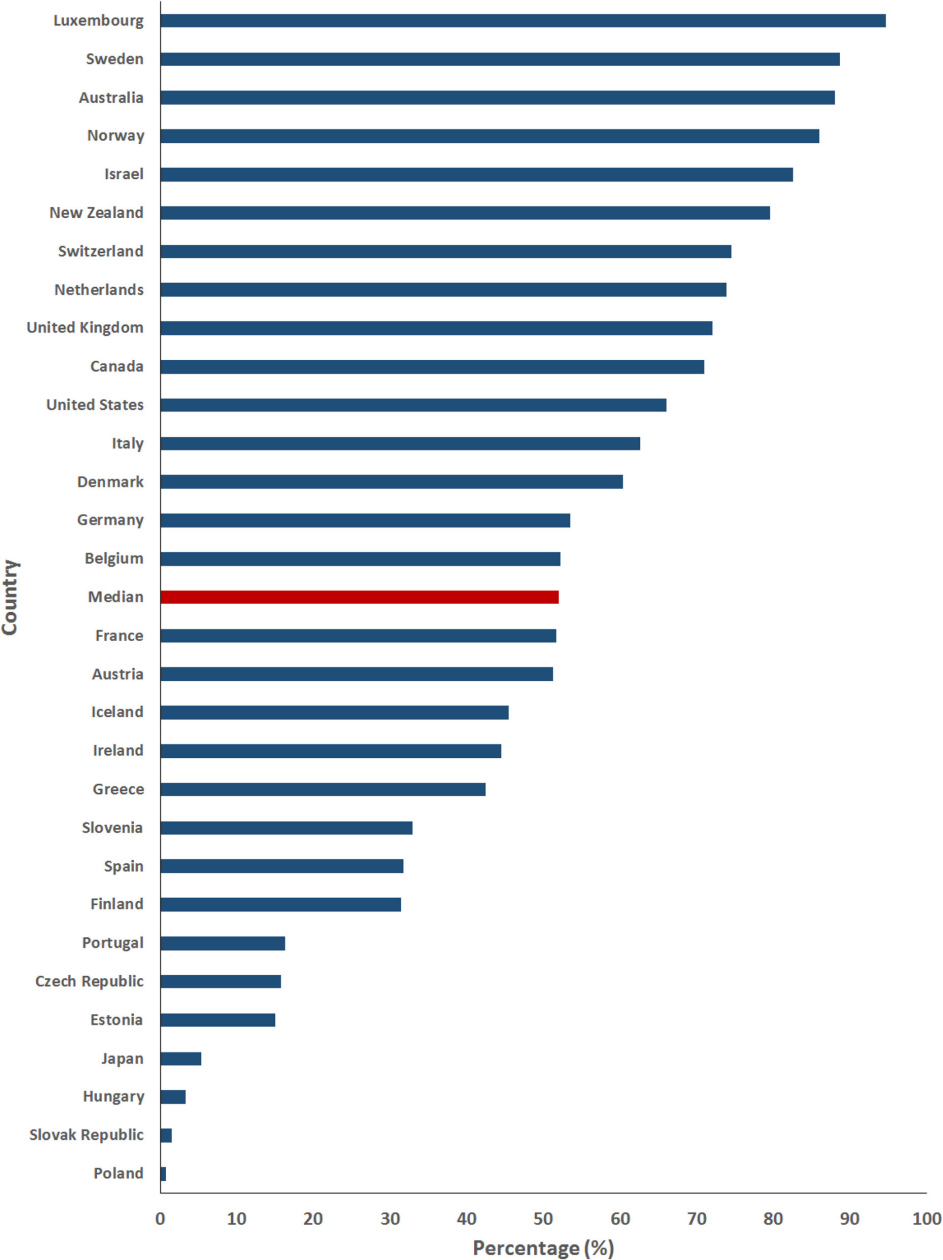
Percentage of tuberculosis notifications in the foreign-born for selected OECD high-income countries

Yet the overall changes seen in TB incidence in high-income countries hide an important disparity: while local-born cases have remained static or decreased, foreign-born cases have decreased more slowly or increased. From 2000 to 2013, local-born TB cases decreased by half (median 51.3 %; IQR −64.3 – -35.3 %) whilst foreign-born case notifications increased marginally (median 2.3 %; IQR −36.7 – +40.4 %) [5–10]. In just under half of the high-income OECD countries foreign-born TB cases increased [5–10]. Consequently foreign-born individuals, in 2013, made up over half of all TB cases (median 52.0 %; IQR 31.4–73.9 %; Fig. 1) [5–11] with incidence rates 8.7-18.4 times that seen in the local-born population [7, 9, 10, 12].

Drilling down further into the patterns TB notifications in the foreign-born population in high-income countries reveals information about key risk groups with the highest incidence and risk of active TB following migration: migrants from Asia and Africa where the burden of TB is moderate/high, recent migrants (within 5 years of arrival), refugees and individuals with comorbidities (such as HIV infection and diabetes mellitus) [7, 9, 10, 12, 13].

## Migration and reactivation of latent TB infection: key drivers of tuberculosis in migrants in high-income countries

### Understanding the scale and nature of migration to high-income countries

In 2013, United Nations figures showed that the global number of migrants was 232 million – a 50 % increase over the preceding two decades [14] with a concomitant change in the pattern of sending countries [15] as globalisation, conflict, and financial reasons have become increasingly important drivers of migration flows. This has resulted in more permanent migrants moving from low/medium income, higher TB burden, countries to high-income developed, lower TB burden, countries including USA, Canada, Australia and Western European nations although migrants are not necessarily representative of the population in the country of origin [15]. Top migrant sending countries for each high-income country will vary according to historic, linguistic, cultural links and geographic proximity. In the UK a significant proportion of the foreign-born migrants arrive from former colonies in Sub-Saharan Africa and the Indian Subcontinent whereas in the US the majority of the foreign-born population originate from Central and South America [16]. As a consequence of migration, in high-income OECD countries, the median proportion of the population that is foreign-born is estimated to be 13.7 % (IQR 10.8-18.4 %) [17].

### Reactivation of latent TB infection in determining TB burden in migrants

**Fig. 2:**
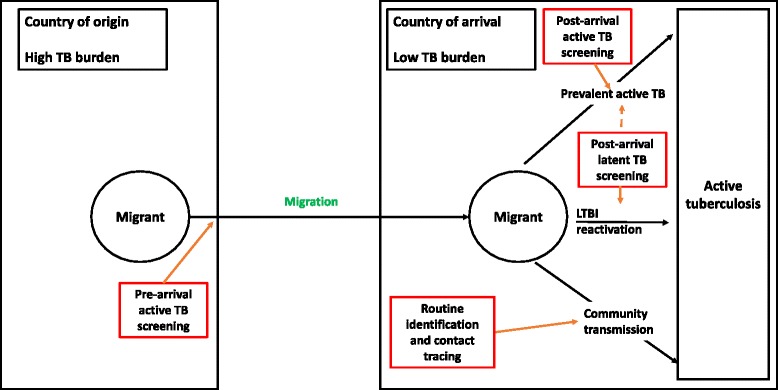
Schematic diagram of migration, factors determining how incident active tuberculosis occurs and methods of screening migrants. Footnote: As a by-product of post-arrival latent TB screening, some cases of prevalent active TB may be identified

The TB burden observed in foreign-born individuals occurs due to one of three reasons: (1) migrants from overseas must either have active TB on arrival, (2) migrants have remotely-acquired latent TB infection which reactivates post-arrival or (3) migrants acquire TB, following arrival, through local transmission (Fig. 2).

#### Active TB disease in migrants on arrival in the receiving country

Surveillance data, and findings from previous meta-analyses (Table 2), have shown that the proportion of migrants with active TB present at the time of migration is relatively small (0.35 %) [18, 19].

**Table 2:** Yields for active tuberculosis from previous meta-analyses

Author	Year	Yield for active tuberculosis (%)		
		Overall	Pre-arrival	At/post-arrival
Klinkenberg [19]	2009	0.35	1.21	0.31
		0.51		
Arshad [18]	2010	0.35	-	0.35
Aldridge [71]	2014	0.22	0.22	-

#### High prevalence of latent TB infection and risk of progression to active disease

Latent TB prevalence figures in migrants are primarily derived from cross-sectional studies where, depending on the specific population tested and the diagnostic tool used, 5-72 % of migrants test positive for LTBI [20–43]; this is independently associated with increasing age and TB incidence in country-of-origin [41, 42, 44]. One can therefore infer that it is both the cumulative duration of exposure and the TB burden in the source country which determines whether individuals will have LTBI [29, 41, 42, 44, 45]. Migrants with LTBI are coming to lower incidence settings and in the initial years following arrival in the destination country, have a higher risk of LTBI reactivation which decreases slowly over time but remains higher than rates in the host population [12, 46–48] This higher rate of reactivation in the initial one to two years after migrants arrive likely reflects latent tuberculosis infection which has been acquired in their country of origin shortly before migration although there is also likely to be an ongoing complex interplay, in the destination country, of host (such as age, and comorbidities including diabetes mellitus) and environmental factors (such as nutritional status) which contribute to the observed epidemiology. Understanding the natural history of TB in recently arrived migrants is important when we are considering how best to implement TB control in this population.

Whilst the literature has expanded rapidly in respect of cross-sectional data on LTBI prevalence, longitudinal data on the risk of migrants with diagnosed, untreated, LTBI progressing to active TB disease remain limited partially due to low numbers of chemoprophylaxis-naïve patients (due to recommendations to treat, and not withhold treatment from, individuals identified with latent TB such as in the UK) and because studies to answer this research question need a large sample size and long duration of follow-up. A UK study followed migrants, predominantly from the Indian Subcontinent, and found a TB progression rate of 16.3 % amongst untreated tuberculin skin test (TST) positive patients over a 15 year period following UK arrival [49]. The risk was significantly higher for young migrants (aged 16–19) and for women [49]. However this study did not adjust incidence rates for travel back to countries of origin nor underlying medical comorbidities. A Norwegian study of asylum seekers found a progression rate of 1.1 % over a follow-up period of up to 32 months [50]. By contrast, Marks and colleagues evaluated a large cohort of predominantly Southeast Asian migrants, with no evidence of active TB, over a mean follow-up of 10.3 years post-arrival in Australia and found that 0.12 % with a positive TST progressed to active TB disease per year [51]. Further work is needed to determine both the overall risk of progression from LTBI to active TB disease in migrants as well as the contribution of concomitant medical co-morbidities and demographics such as diabetes mellitus, chronic kidney disease and age. Diabetes mellitus and chronic kidney disease are more common in migrant populations and significantly increase the risk of reactivation from LTBI to active TB [13, 52]. This is likely to result in increasing TB notifications in the foreign-born, migrant populations, particularly as the migrant population ages, and this will, therefore, need to be taken into consideration when developing TB control programmes [13].

An additional issue which is likely to play a part in determining TB epidemiology in migrant populations is the acquisition, and subsequent reactivation, of LTBI following re-exposure during travel back to their countries of origin. Previous work in this area has indicated that travel to high TB burden countries increases the risk of acquiring LTBI with the risk increasing with more prolonged travel and a higher TB burden in the country visited [53]. Although there is a paucity of data on the proportion of TB acquired through travel, published work suggests this could be anywhere between 20 % and 50 % [54, 55]. However further prospective research in this area is needed to more accurately quantify the risk.

#### Using molecular genotyping to distinguish reactivation of latent TB and recent transmission of active TB in migrants

In order to distinguish reactivation of remotely acquired LTBI from TB transmission, authors have genotyped DNA isolated from *M. tuberculosis* cases in foreign-born individuals. Here, individuals with a TB isolate with a clustered DNA pattern that matches at least one other case in the cohort is attributed to recent transmission whereas cases with unique, DNA patterns are thought to arise due to the reactivation of LTBI [56, 57]. In a large meta-analysis across a range of TB burden settings, foreign-born individuals were significantly less likely to have a clustered isolate as compared to local-born individuals (25 % versus 45.8 % clustered respectively) [57]. Moreover, there is little evidence that the foreign-born population transmit TB to the local born population [58]. These data, in conjunction with TB surveillance data, appear to highlight the importance of reactivating LTBI amongst migrants in determining TB epidemiology in high-income countries and, therefore, the need to have appropriate control measures in place.

## Tuberculosis control with a special focus on migrants

Globally, TB control policies focus on quickly diagnosing and treating individuals with active TB. Additionally in high-income countries this is complemented by the contact-tracing of household contacts of smear-positive cases with the overall aim of reducing onward transmission. However this method of TB control does not fully address the potential source of reactivation of remotely acquired LTBI progressing to active TB disease – such as that seen in migrants. Dynamic transmission models have been used to study the impact of chemoprophylaxis for LTBI on global TB control and concluded that concurrently targeting individuals with active TB disease and individuals with LTBI will augment TB control [59–63]. The growing importance of tackling LTBI is reflected by recent WHO guidelines which conditionally recommend migrants from high TB burden countries are offered screening and LTBI treatment [64].

### Migrant screening practices and their outcomes in high-income countries

Several authors have recently reviewed the migrant TB screening programmes in high-income countries. Each program is different and they differ by whether screening is done for active or latent TB (or both), when screening is performed in relation to arrival in the host country, which groups of migrants are screened (refugees or other migrants groups; which countries of origin) and which tools are used to screen for active and latent TB (see Table 3) [65–69]. It should, however, be borne in mind that much of the available data relates to documented migrants and there remains ongoing difficulty in collecting data on undocumented migrant who bypass standard screening protocols.

**Table 3:** Potential strengths and weaknesses of different migrant screening methods

	Screening methodology	
	Screening for active tuberculosis	Screening for latent tuberculosis infection
Screening tool used	Chest x-ray	Tuberculin skin test
		Interferon gamma release assay
Screening location	Pre-arrival	Post-arrival
	At arrival	
	Post-arrival	
Strengths	Able to identify active TB	Identifies latent TB before reactivation occurs
	Able to identify infectious individuals	Can be built into community programmes
	Can be integrated into immigration processes	Targeted screening likely to be cost-effective
Weaknesses	Low yields for active TB	Programmatically difficult to implement
	Uncertain cost-effectiveness (unless screening targeted)	Numbers accepting and completing treatment may be suboptimal
	Does not identify patients with latent TB who can go on to reactivate	

#### Screening practices for active tuberculosis

Most OECD high-income countries screen migrants for active TB although the specifics of how this is performed vary significantly across countries. Most active TB screening programs in Western Europe are performed on or soon after arrival with a chest radiograph (CXR). Other countries such as Canada, the US, Australia, New Zealand and recently the UK, screen for active TB with a CXR prior to arrival. If the CXR is abnormal a sputum smear and culture are performed. Those found to have active TB are treated prior to arrival and granted permission to enter the country if cultures are negative at the end of treatment. Those found to have an abnormal CXR but negative sputum cultures or those with prior treated TB are followed after arrival in a post-landing surveillance program [65–70].

The criteria for which migrant groups are screened is also highly variable [67, 69]. Alvarez and colleagues found that countries differed in which migrants were selected for screening basing their decision on a number of factors including: type of migrant (e.g. refugee, students, workers), duration of stay, intended occupation or TB burden in country of origin [67]. In a large survey of OECD countries, the authors identified heterogeneity in source country incidence thresholds for screening although, in general, migrants arriving from high TB burden settings were preferentially selected for screening [69]. The reasons for this remain unclear but it may reflect a lack of evidence in this area.

#### Outcomes of screening for active TB

Whilst screening for active TB is frequently undertaken, there is a lack of trial data for its effectiveness as a public health intervention. Policy decisions therefore rely on national evaluations and cross-sectional observational data.

Pre-arrival screening, in country of origin, is part of the immigration process of several high-income countries – including the US, Australia, Canada and the UK. Aldridge and colleagues undertook a detailed systematic review and meta-analysis on the yields of active TB through pre-arrival screening. They found that the overall yield for culture positive active TB was 0.22 % (219 cases/100,000) [71]; the yield for culture positive TB increased with increasing TB prevalence in country of origin [71] suggesting that setting an incidence threshold for pre-arrival screening of migrants may be needed to ensure cost-effective use of resources. Klinkenberg et al. reviewed the yields for active TB at different stages of the immigration process and found that the yields for pre-arrival, as compared to post-arrival, screening were higher (1.21 % versus 0.31 % respectively) although these data were based entirely on non-EU studies [19]. A recently published analysis of the US pre-arrival screening programme found that 4032 cases of culture positive TB were diagnosed amongst 1,561,460 migrants screened (yield 0.26 %) resulting in a reduction in the number of TB cases diagnosed amongst migrants within one year of arrival in the US [43]. The UK pre-arrival screening programme yield for active TB has steadily increased from 0.05 % in 2006 to 0.16 % in 2014 which most likely reflects the use of sputum cultures [72, 73].

Post-arrival screening for active TB involves chest radiography once the migrant has arrived in the host country. Two systematic reviews have evaluated the outcomes and yields for active TB in high-income countries [18, 19]. Arshad et al. reviewed 22 studies comprising 2,620,739 migrants and found that the overall yield for post-arrival screening was 0.35 % with higher yields in migrants from Africa and Asia [18]. Klinkenberg and colleagues reviewed 40 studies and found that the yields for post-arrival screening, which were lower than that for pre-arrival screening, ranged from 0.20 % to 0.36 % depending on the specific setting in which screening was undertaken [19].

Previous work has shown that the current models of active TB screening have weaknesses including: individuals not completing the screening processes, limited yields for active disease and an inability to identify active TB occurring through LTBI reactivation [13].

#### Screening practices for latent tuberculosis

Whilst most countries offer some form of screening for active TB, screening for LTBI is much less commonly performed [69]. High-income countries that screen for LTBI usually undertake this post-arrival with a tuberculin skin test (TST) or an interferon gamma release assay (IGRA) [69, 74].

For reasons of practicality and cost-effectiveness, most high-income countries attempt to limit the eligible population to refugees or asylum seekers or those individuals arriving from high TB burden settings [69]. However, countries vary considerably in their definition of a high TB burden setting for the purposes of migrant screening [69]; the UK has taken a decision to screen migrants arriving from countries in Sub-Saharan Africa or those countries with a TB incidence above 150 per 100,000 whereas Canada screens at a lower threshold of 30/100,000 but only migrants with increased risk of reactivation. Given the prevalence of comorbidities in the migrant population (such as diabetes mellitus,) which increase the risk of reactivation, these factors will likely need to be taken into account when determining which migrants to screen. At the present time, however, the variation between guidelines could reflect uncertainty about the optimal screening threshold which balances the need to identify the majority of LTBI with cost effectiveness.

#### Outcomes of screening for latent tuberculosis infection

Successful screening for LTBI involves a number of key interlinked steps including the accurate identification of migrants, appropriate screening of migrants, initiation of chemoprophylaxis and completion of therapy. However most of the data on LTBI screening outcomes come from cross-sectional studies which have been conducted with the primary aim of calculating the prevalence of LTBI in migrants (estimated at around 25-30 % in young adult migrants from high incidence countries) [20–43]. However, there is less data on the other elements of the screening pathway – including uptake, and completion, of chemoprophylaxis. A Canadian group recently reviewed the data on LTBI screening effectiveness and found that migrants dropped out at each step of the screening pathway so that overall only 31 % of the cohort completed the programme successfully highlighting the need for research into interventions to optimise the LTBI screening pathway [75]. Programmatically, at a national level, there is little observational data on the impact of LTBI screening on TB notifications in migrants although the recently commenced UK national migrant screening programme will provide this useful data in the next few years [76].

### Health economics of migrant screening

Whilst the programmatic outcomes of migrant screening are important, a key consideration for policy-makers and clinicians is cost-effectiveness. Several studies have explored the cost-effectiveness of migrant screening for TB although they have focused on different aspects of screening including whether to screen for active or latent TB, which migrant groups to screen and how to screen [41, 42, 77–86].

#### Cost-effectiveness of screening for active and latent tuberculosis

Screening migrants for active TB is widely implemented by high-income countries albeit with different models of care. However there are few studies formally examining the cost-effectiveness of this intervention. Previous studies have come to differing conclusions about the cost-effectiveness of screening migrants for active TB. Schwartzman and colleagues modelled the comparative cost-effectiveness of migrant screening using chest radiography versus tuberculin skin test (versus no screening) and found that in migrants with a high prevalence of infection that chest radiography was the most cost-effective screening modality although the TST strategy prevented the most cases of TB. By contrast, Dasgupta et al. constructed a Markov model informed by empirical data to compare the cost effectiveness of screening migrants with chest radiography pre-arrival followed by TST (if the CXR showed any abnormalities) with screening close contacts of index sputum smear-positive cases (with TST followed by CXR) [77]. The authors found that migrant screening using chest radiography was not cost-effective due to difficulties with operationalising screening [77]. The lack of research in this area highlights the need for further health-economic analyses to objectively assess, and make conclusions about, the cost-effectiveness of screening migrants for active TB.

A larger number of published studies from high-income countries have focused on evaluating the cost-effectiveness of screening migrants for LTBI [78–86]. These studies, which have evaluated different aspects of LTBI screening including which migrants to screen and how to screen, have generally concluded that LTBI screening of migrants from high burden countries, mainly Asia and Africa is a cost-effective intervention in high-income countries [41, 42, 78–86].

Methods for diagnosing LTBI have evolved over the last decade with IGRAs increasingly replacing the TST [87]. This is reflected by the several studies which have explored the relative cost-effectiveness of different screening modalities and algorithms for LTBI [41, 42, 79–84]. These health-economic analyses have, in general, found that IGRA are more cost-effective than TST [41, 42, 79, 81, 84]. However, in the absence of robust longitudinal data as for TST, there remains ongoing debate about the use of IGRA as a screening tool with certain national guidelines instead advocating the use of TST [13].

#### Limitations of cost-effectiveness studies

The scarcity of randomised clinical trials has meant that policy decision are mostly based on of health-economic modelling and observational data. However models must, by definition, make a number of simplifying assumptions and their results are therefore highly dependent on model structure and specific model parameters – even if there is uncertainty around these due to a lack of empirical data.

## Future directions

The impact of migration on tuberculosis epidemiology in high-income countries is increasingly well-recognised and there has been a shift towards augmenting TB control by screening migrants for TB. Up until recently there has been a lack of coordinated international guidance in this area but the European Centre for Disease Prevention and Control (ECDC) is currently formulating guidance on the screening of migrants for a range of infectious diseases – including TB [88]. As migrant screening for TB becomes more embedded into TB control programmes, there will be an increasing need for high-quality operational research to establish how to undertake TB screening most effectively and integrate it with migrant health programmes including testing for blood-borne viruses.

## Conclusions

In this review we have comprehensively brought together the literature with respect to all aspects of tuberculosis and migration. Tuberculosis in high-income countries continues to be a cause of morbidity and mortality – particularly amongst individuals who have been born overseas in high TB burden, low-income countries and migrated to high-income countries. The reasons for the burden of disease in the foreign-born, migrant, population are primarily due to migration from high TB burden settings and the reactivation of remotely-acquired latent TB infection. As a consequence there is increasing focus on how best to enhance TB control through the coordinated screening of migrants for TB. Whilst most countries focus on screening migrants for active TB, this has a relatively low yield on its own and it is likely that the most effective and cost-effective means of screening migrants for TB will comprise multiple, inter-linking elements: pre-arrival screening for active TB and targeted post arrival screening for LTBI in migrants from intermediate/high TB burden settings. However, the programmatic implementation of migrant screening is potentially hampered by limited uptake, acceptance and completion of therapy. There is an urgent need for further coordinated research in this area to inform future national and international guidance.

### Ethical approval

Ethical approval not required as this is a literature review.
